# Regulon organization of Arabidopsis

**DOI:** 10.1186/1471-2229-8-99

**Published:** 2008-09-30

**Authors:** Wieslawa I Mentzen, Eve Syrkin Wurtele

**Affiliations:** 1 CRS4 Bioinformatics Laboratory, Parco Scientifico e Technologico POLARIS, 09010 Pula (CA), Italy; 2 Department of Genetics, Development and Cell Biology, Iowa State University, Ames, IA 50011, USA

## Abstract

**Background:**

Despite the mounting research on Arabidopsis transcriptome and the powerful tools to explore biology of this model plant, the organization of expression of Arabidopsis genome is only partially understood. Here, we create a coexpression network from a 22,746 Affymetrix probes dataset derived from 963 microarray chips that query the transcriptome in response to a wide variety of environmentally, genetically, and developmentally induced perturbations.

**Results:**

Markov chain graph clustering of the coexpression network delineates 998 regulons ranging from one to 1623 genes in size. To assess the significance of the clustering results, the statistical over-representation of GO terms is averaged over this set of regulons and compared to the analogous values for 100 randomly-generated sets of clusters. The set of regulons derived from the experimental data scores significantly better than any of the randomly-generated sets. Most regulons correspond to identifiable biological processes and include a combination of genes encoding related developmental, metabolic pathway, and regulatory functions. In addition, nearly 3000 genes of unknown molecular function or process are assigned to a regulon. Only five regulons contain plastomic genes; four of these are exclusively plastomic. In contrast, expression of the mitochondrial genome is highly integrated with that of nuclear genes; each of the seven regulons containing mitochondrial genes also incorporates nuclear genes. The network of regulons reveals a higher-level organization, with dense local neighborhoods articulated for photosynthetic function, genetic information processing, and stress response.

**Conclusion:**

This analysis creates a framework for generation of experimentally testable hypotheses, gives insight into the concerted functions of Arabidopsis at the transcript level, and provides a test bed for comparative systems analysis.

## Background

Genes that share a similar expression profile across multiple spatial, temporal, environmental and genetic conditions are likely to be under common transcriptional regulations. Such sets of coexpressed genes could be considered eukaryotic regulons [1]. Meta-analysis of microarray data, sometimes combined with other types of data – proteomics, co-precipitation, literature, yeast two hybrid – has proven valuable for model organisms including bacteria [2], nematode [3], human [4,5], chimpanzee [6], mouse [7], rat [8] and yeast [9-11]. The use of transcriptome data alone has allowed for identification of functionally coherent modules corresponding to major cellular processes in yeast [12-14] and some of these modules might be important enough to be conserved across eukaryotic organisms [12].

Meta-analysis of the Arabidopsis transcriptome thus offers the potential to identify prevailing cellular processes, to associate genes with particular biological processes, and to assign otherwise unknown genes to biological processes they are correlated with. Despite the model plant Arabidopsis genome having been fully sequenced since 2000, the function of many of its over 27,000 protein-coding genes is experimentally undetermined. Almost 9,000 of the genes cannot be ascribed any function. Many other genes contain a domain recognizable as representing a general molecular or biochemical function (phosphorylation, glycosylation), but no clear physiological function; i.e., the nature of their involvement in cellular processes is not understood (TAIR8 Genome Release, April 28, 2008 [15]). The effort to assign the function to otherwise unknown genes that are correlated with genes of known function was recently undertaken by Horan and coworkers [16]. The authors used the clustering of expression data to propose a function to 1547 genes coding for proteins of unknown function (PUF) and set up a Plant Unknown-eome Database (POND) [17].

Arabidopsis expression data is available across a wide range of perturbations of nutrients, stress, and light, in the framework of defined organs, genetic backgrounds, and developmental stages. With this wealth of data it is tempting to identify genes that share common expression signatures across a variety of experiments. Thus, the Arabidopsis transcriptome is receiving growing attention, despite the challenges associated with a high volume of genes, distribution of data across multiple databases and publications, and incompleteness of the biological data and metadata. Several online repositories for microarray data and metadata storage and/or analysis have been created, including NASCArrays [18,19], Genevestigator [20,21], PLEXdb [22,23], MetaOmGraph [24], ArrayExpress [25,26], Vanted [27,28], VirtualPlant [29], ATTED-II [30,31], Arabidopsis Coexpression Data Mining Tools [32,33], Bio-Array Resource [34,35], MapMan [36,37], PageMan [38,39] and CressExpress [40,41]. Based on the data from public datasets, coexpression of genes in the indole, flavonoid and phenyl-propanoid biosynthetic pathways has been reported [42]. Similarly, coexpression has been shown for genes encoding synthesis of cellulose and other cell wall components [43-45] and oxidative phosphorylation [46]. Morcuende et al. [47] used large-scale transcript data, along with metabolomic and enzymatic activity data, to investigate finer-tuned changes in Arabidopsis regulatory networks during phosphate starvation. Ma and Bohnert [48] and Weston et al. [49] classified characteristic transcriptome responses to stresses, using public microarray data. Biehl et al. [50] assigned 1590 Arabidopsis nuclear genes, mostly encoding plastid-localized proteins, to 23 regulons, based on RNA accumulation profiles across 101 different conditions. Wei et al. [51] study of the transcriptional coordination of 1,330 genes coding for enzymes in AraCyc pathways [52,53] indicated a broad transcriptional basis for coexpression of metabolic pathways. Recently, Ma et al. [54] presented an Arabidopsis gene coexpression network based on partial correlation analysis. With the random sampling of genes, the authors circumvented the computational problem resulting from small number of samples versus large number of genes and approximated direct associations between the genes, obtaining a network with 6760 genes and 18,000 interactions.

Here, we present a global analysis of the regulon organization of the Arabidopsis genome, derived from the results of the graph clustering of the coexpression network of the 13,456 genes. The relationships among genes in a complex organism clearly entail shifts in alliances among the genes, resulting in "fuzzy" memberships [55] in different clusters according to perturbations in environmental and genetic conditions. None-the-less, this analysis captures the prevailing transcriptional network of the organism. As such, it provides a strategy to evaluate functions of genes in a given gene family, and to develop experimentally testable hypotheses about the functions of genes with no known physiological or developmental role. The approach applied in this study delivers a perspective that is not constrained by existing assumptions about the organization of plant processes. Instead, the organization emerges directly from observations. The analysis reveals biologically coherent functional modules, representing a sometimes surprising combination of metabolic and developmental genes.

## Results

### Graph-clustering of Arabidopsis transcriptome data

**Figure 1:**
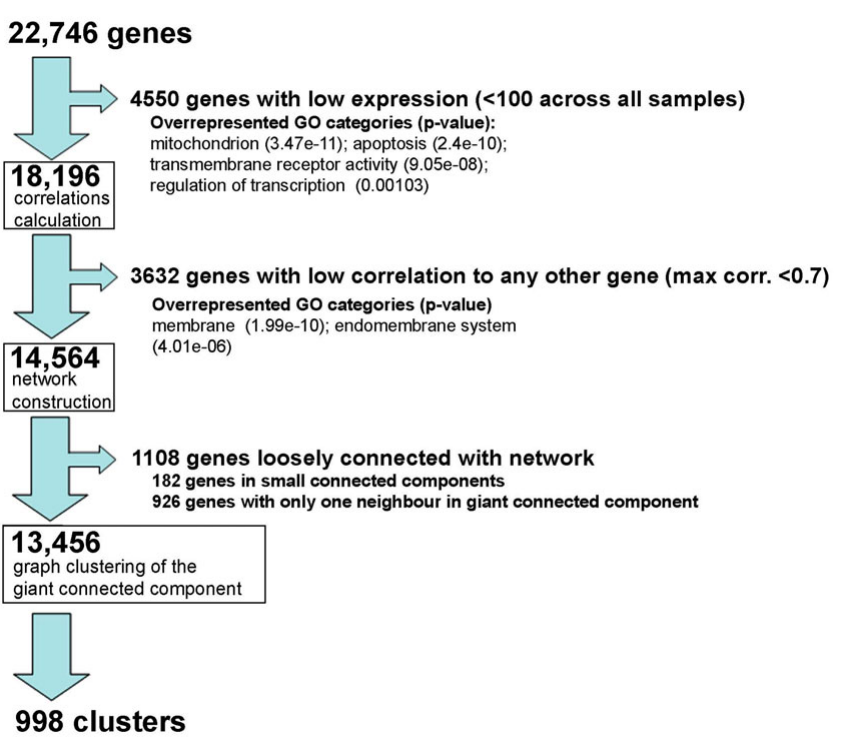
**Data processing for construction of the transcriptional network**. Filters were applied to original probe sets on ATH1 chip to remove the genes with expression lower than the mean of 100 and with correlation to other genes lower than the threshold of 0.7. The network was then constructed and the largest connected component of this network was retained; smaller connected components as well as genes with only one neighbor in the giant connected component were filtered out. This resulting network, containing 13,456 genes, was then clustered. Enrichment of Gene Ontology terms in groups of filtered genes is indicated.

To facilitate identification of the regulon organization of coexpressed Arabidopsis genes that reflect the most prevailing processes in this plant, we performed meta-analysis of multiple microarray experiments. We developed a transcriptome data set for 70 experiments from the public microarray depositories NASCArrays [19,18] and PLEXdb [23,56] (described in detail in Methods). The experiments from these databases incorporate a wide variety of mutants, environmental conditions and stages of development. To avoid artifactual signals, samples and experiments with poor replicate quality were removed. The resulting 963 chips were normalized to the common range and the replicates were averaged to yield 424 samples. To further minimize noise in the data, probe sets with low expression values (defined as probes whose expression in every microarray chip was lower than the mean value for that chip) were removed from analysis (Figure 1). In order to concentrate on the most prominent coexpressed sets, only genes correlated above Pearson's correlation threshold of 0.7 with at least two other genes were included in the analysis. The expression data for the resulting 13,456 probe sets was treated as a coexpression network in which genes are represented as nodes, and two nodes are connected by an edge if the Pearson correlation between their RNA accumulation profiles is higher than a threshold of 0.7.

Pearson's R has been chosen as the similarity measure between the expression profiles, in spite of its known shortcoming – measuring the strength of only linear relationships and sensitivity to outliers. We estimate that the presence of strong non-linear relationships between gene expression profiles in expression data, which would not be picked up by Pearson's R, is relatively rare. The results presented by Daub et al. [57], where authors found no increase in the discovery of high correlations in gene expression data when using measures of non-linear relationships (mutual information) agree with this assertion. Pearson correlation gives high score for the expression profiles which consist of mostly very low values and one, or few, very high values, which in our dataset often occurs for genes that are expressed only in couple of underrepresented tissues or conditions. This sensitivity to outliers is usually seen as the drawback of Pearson's correlation measure. However, we decided that for the purpose of our analysis these outliers are valid, though extreme, datapoints and that clusters based on the presence of genes in only couple of samples do represent valid clusters. For the groups of genes with expression profiles that are variable and parallel across many diverse conditions, a hypothesis of common regulatory program acting upon participating genes is particularly plausible. For clusters that are active only under a small subset of conditions, there is less ground for a co-regulation assumption. However, such clusters may also reveal valuable information. For example, the subset of genes for increased growth in response to auxin and cytokinin upregulated only in cell cultures and tumors would be hard to pinpoint if not for meta-analysis. Correlations based on extreme values would not be found with Spearman rank correlation, Kendall's tau or in logged data.

We aimed to identify sets of coexpressed genes, which are represented in our model as densely-connected regions of the network. A graph-clustering method based on flow simulation (Markov chain graph clustering, MCL) was used to identify clusters in this network that correspond to the sets of coexpressed genes. This method, developed by van Dongen [58], has been used previously for clustering protein sequence data [59] and for identifying modules in the yeast protein interaction network [60]. One of the advantages of Markov clustering is that it is scalable to large graphs, unlike most other graph clustering algorithms, which are not applicable to graphs with more than 5000 nodes. Using MCL, we identified 998 clusters in the Arabidopsis network, ranging in size from 1 to 1623 genes.

**Figure 2:**
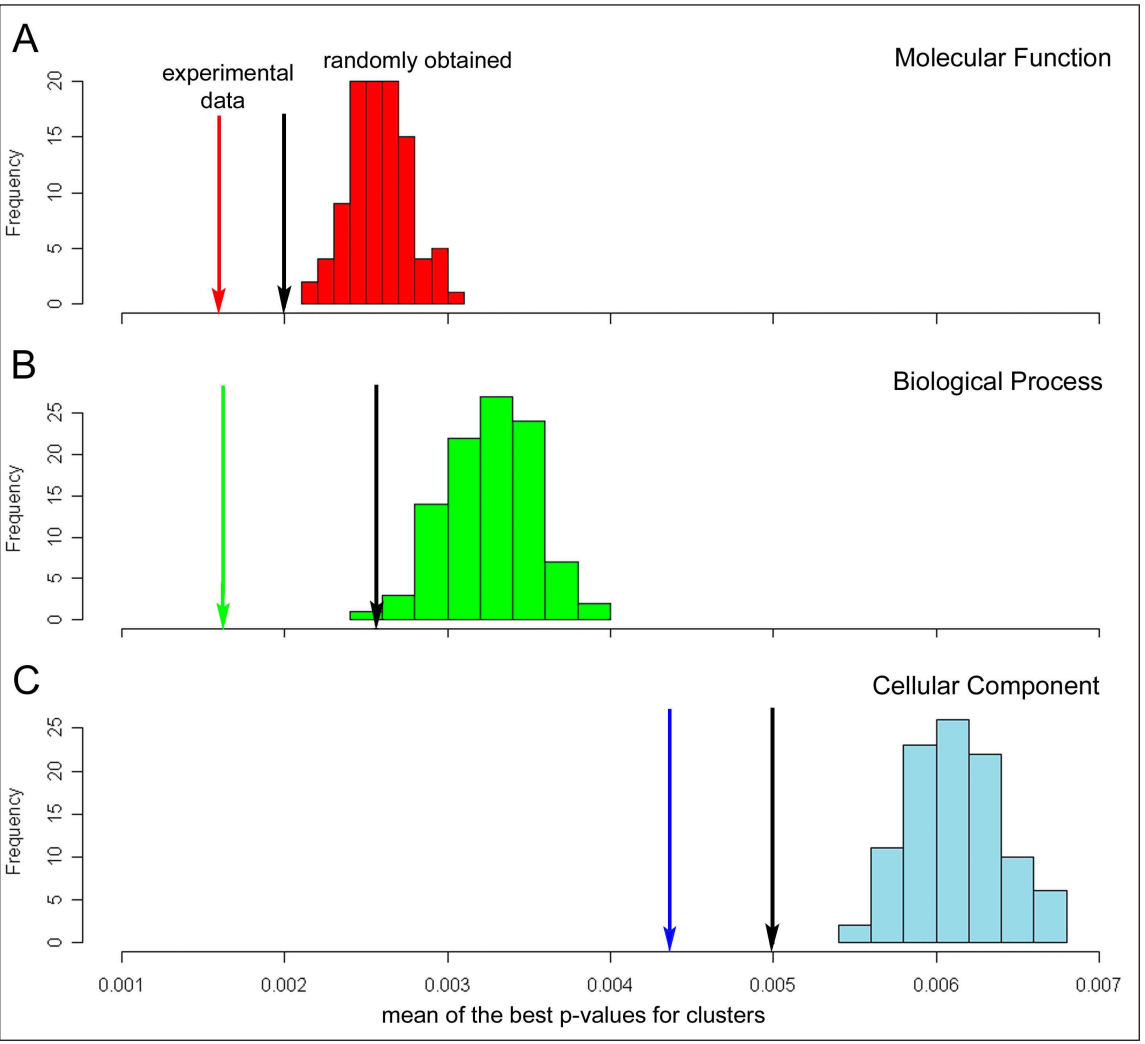
**Statistical significance of Markov chain graph clustering results**. The best p-values for over-representations of Gene Ontology (GO) terms, averaged over all clusters (S score, denoted by color arrow) are compared to the analogous values for 100 randomly-obtained clusterings (histogram). GO categories: (**A**) Molecular Function, (**B**) Biological Process, (**C**) Cellular Component. In each case, the actual clustering scored significantly better than any of 100 randomly obtained ones (Wilcoxon test p-value < 2.2 × 10^-16^). In a comparison of the MCL (Markov clustering) and k-means clustering results (the latter denoted by black arrows), MCL had better S scores for GO terms overrepresentation than the k-means method (0.0016 versus 0.0020 for "Molecular Function" category; 0.0016 versus 0.0026 for "Biological Process"; and 0.0044 versus 0.0050 for "Cellular Component").

To evaluate the significance of the clustering results, we compared the overrepresentation of Gene Ontology (GO [61]) terms in the set of the 148 largest regulons (i.e., containing at least 10 genes) derived from Markov clustering of the experimental data, with GO terms overrepresentation of 100 randomly-obtained sets of clusters. For each randomly-obtained set, clusters were designated by permuting the gene locus IDs, such that the sizes of the 148 clusters were preserved relative to the experimental data, but the genes assigned to each cluster changed (see Methods section). The best p-value for overrepresentation of GO terms was recorded for each cluster in a set and averaged over all clusters. Distribution of p-values for GO terms in the randomly-obtained clusters was then compared to the respective value for the regulons derived from the experimental data (Figure 2). For each GO terms category (Molecular Function, Biological Process, Cellular Component), the experimental dataset scores significantly better than any of the randomly-obtained sets (Wilcoxon test p-value < 2.2 × 10^-16^). This analysis indicates that the concentration of similar GO terms in the clusters derived from experimental data is not random, and thus these regulons might correspond to meaningful biological processes.

The Markov clustering result was also compared with results obtained from another common method, k-means clustering, with the same number of clusters (= 998) as a parameter. The clusterings produced by MCL and k-means differed in the distribution of the cluster sizes, which might have influenced their scoring. The agreement between gene assignments to regulons by these two methods is 0.044 (based on the adjusted *rand *index; as compared to an agreement between MCL clustering and random reassignment of genes to clusters of only 10^-5^). MCL clustering yielded somewhat better S scores for GO terms overrepresentation than the k-means method (0.0016 versus 0.0020 for the Molecular Function category, 0.0016 versus 0.0026 for Biological Process category, and 0.0044 versus 0.0050 for Cellular Component category). MCL clustering had also higher Z-score for the total mutual information between the clustering and all the GO terms describing the genes within the clustering (80.1 versus 54.2 for k-means clustering).

The prevalent physiological or developmental functionality of each regulon containing over 20 genes (69 regulons comprising collectively 9,436 genes) was examined in more detail; the results are summarized in Table 1. To assign functionality to a regulon, we combined the results from two independent methods, automatic analysis of enrichment of GO terms and manual inspection of annotation supplemented by literature searches for each gene's annotation and function, as well as examination of the conditions under which the genes of the regulon are maximally expressed. Most regulons are characterized by a mixture of molecular functions (enzymes, transporters, transcription factors and signaling molecules) that work together to achieve a common goal. This goal could be, for example, hormone-mediated development of floral organs accompanied by metabolic processes (Regulon 43), or a defense response leading to synthesis of protective compounds (Regulon 46). One cluster is almost exclusively devoted to proteolysis (proteasome complex in Regulon 45). Although genes with low expression have been filtered out, the genes that predominate in three of the larger clusters (Regulons 11, 17, and 58) are annotated as "hypothetical", transposons", or "pseudogenes".

**Table 1:** Predominant functions of the regulons

**Regulon**	**# of genes**	**Postulated physiological function**	**FC^a^**
1	1629	mixed (tricellular and mature pollen-specific) ^b^	ND
2	1136	Photosynthesis	29
3	869	protein synthesis	65
4	583	Mitosis	49
5	507	membrane transporters -metal, toxins removal (root-preferential)	77
6	417	embryo maturation (fruit and seed-preferential)	25
7	330	developmental regulation (leaf apex- preferential)	38
8	281	information (uninucleate microspore and bicellular pollen-specific)	64
9	234	response to environmental stimuli	26
10	223	protein modification, defense response	66
11	215	nuclear, others with very low expression	ND
12	182	mixed (fruit-preferential)	ND
13	154	upregulated in 'response to CO_2 _levels' experiment	ND
14	140	regulation of organ development	61
15	138	plastid stress and circadian rhythm	56
16	121	Information	58
17	115	Information	51
18	100	cell wall, respiration/catabolism (pollen-specific; highest in tricellular pollen)	46
19	96	mixed (flower-preferential)	ND
20	94	Information	80
21	92	secondary products, secondary wall (flower-specific, mostly tapetum)	54
22	81	cell wall biosynthesis, carbohydrate metabolism	47
23	77	membrane proteins	69
24	71	defense response	70
25	70	defense response	77
26	68	Information	79
27	68	regulation, root (root- and hypocotyl-preferential)	73
28	66	nucleic acid binding, regulation	60
29	63	aerobic respiration in mitochondria	92
30	56	Signalling	89
31	52	defense response	25
32	48	nuclear genes, RNA processing, DNA replication	70
33	48	chloroplast organization and biogenesis	62
34	47	mitochondrial genes	96
35	45	kinases, signaling, disease resistance	69
36	43	lipid modification and cuticular wax synthesis (flowers and shoot apex-specific)	54
37	42	heat shock response	60
38	40	RNA processing, translation, transcription regulation	82
39	40	catabolic processes deriving energy	51
40	40	transcription, translation, protein folding and transport	86
41	36	regulation, information	83
42	34	Regulation	78
43	33	flower/fruit, cell wall depositions (flower/fruit-preferential)	48
44	31	metabolic processes in flowers/fruit (flower/fruit-specific)	22
45	30	proteasome complex	87
46	29	defense response	50
47	29	nuclear, replication, chromosome organization, cell cycle	67
48	28	cell culture and tumor specific	ND
49	27	chloroplast-encoded	100
50	27	Signalling	90
51	26	organ specification in shoot (leaf apex- and hypocotyl-preferential)	35
52	26	endoplasmic reticulum: protein folding and secretion/redox function	73
53	25	fatty acid biosynthesis	83
54	23	protein degradation and lipid modification	59
55	23	epidermal/cuticular deposits	43
56	22	nectaries/carpel specific function (carpel-specific)	29
57	22	phloem specific (vasculature tissues-specific)	26
58	22	transposases, mostly CACTA-type	100
59	21	metabolism and transport of triterpenoids (root hairs-preferential)	71
60	21	ubiquitin ligase	53
61	20	metabolism of glutathione and glutamate, redox	56
62	20	information, nuclear	78
63	20	stress-induced catabolism, mediated by jasmonic acid	72
64	20	information, nuclear	87
65	20	secondary metabolism/pathogen infection	61
66	20	exocytosis	29
67	20	Ca ^2+ ^– triggered exocytosis (pathogen response?)	60
68	20	shoot meristem development and nucleic acid binding (leaf apex and hypocotyl – preferential)	69
69	20	leucine/glucosinolates metabolism	65

Regulons with 20 or more genes are shown. Annotations are postulated based on GO terms supplemented with information from the published literature.

^**a **^Functional Coherence, calculated as percentage of annotated genes whose TAIR annotation is consistent with the cluster functional classification. (Genes designated "hypothetical" or "unknown" are not included in this calculation.)

^**b **^prevalent locations of expression are indicated in parenthesis. "specific" refers to virtually all expression in the given location; "preferential" refers to most expression being in the given location

**Figure 3:**
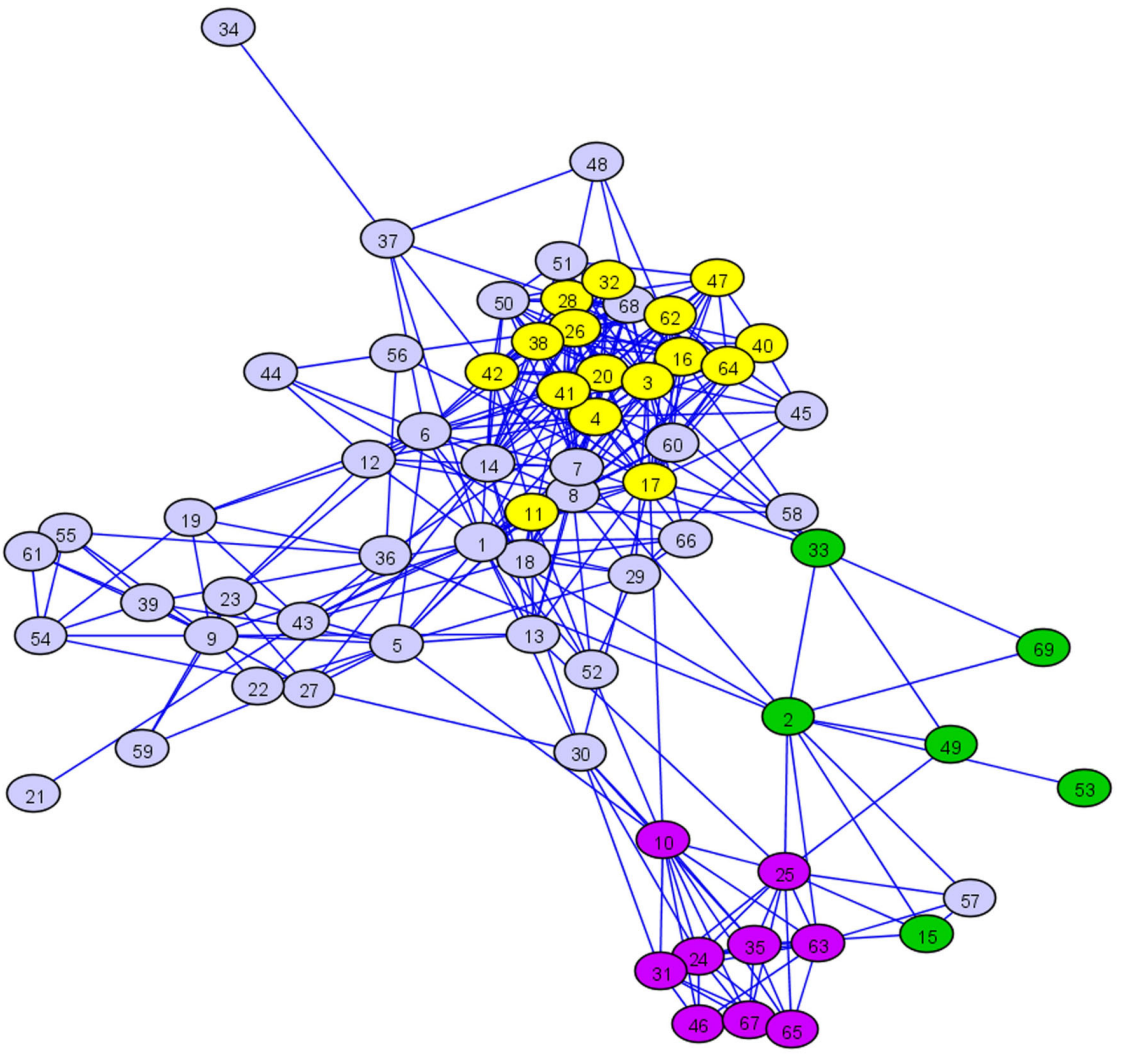
**Higher-order structure in the coexpression network**. All regulons containing at least 20 genes are depicted; these comprise a total of 9,436 genes. Regulons are represented by ovals numbered 1 through 69. A linkage between two clusters means that one or more genes in one of the clusters are correlated with one or more genes in the other cluster. As observed from the proximity of regulons with similar broader functional category, three super-clusters of regulons are revealed: regulons related to information-related functions (purple), plastidic functions (green) and defense response-related functions (yellow). The predominant functionality of each regulon is defined in Table 1. Network was visualized using the GraphExplore tool [118].

A simplified view of the coexpression network formed by 69 largest regulons is shown in Figure 3. A link between two regulons means that there are genes in one regulon that are correlated with genes in the second regulon. It is interesting to note the higher-order grouping of the regulons that predominantly contain genes with genetic information-related, photosynthetic/plastidic, and stress response functions.

### Six regulons are devoted to nuclear-encoded plastidic functions

Six of the regulons with over 20 genes represent plastidic functions that are encoded predominantly by nuclear genes (Regulons: 2, photosynthesis/chloroplast biogenesis; 15, plastid stress and circadian rhythm; 33, plastid organization and biogenesis; 49, plastid-encoded genes; 53, fatty acid biosynthesis; 69, glucosinolate biosynthesis; Table 1).

**Figure 4:**
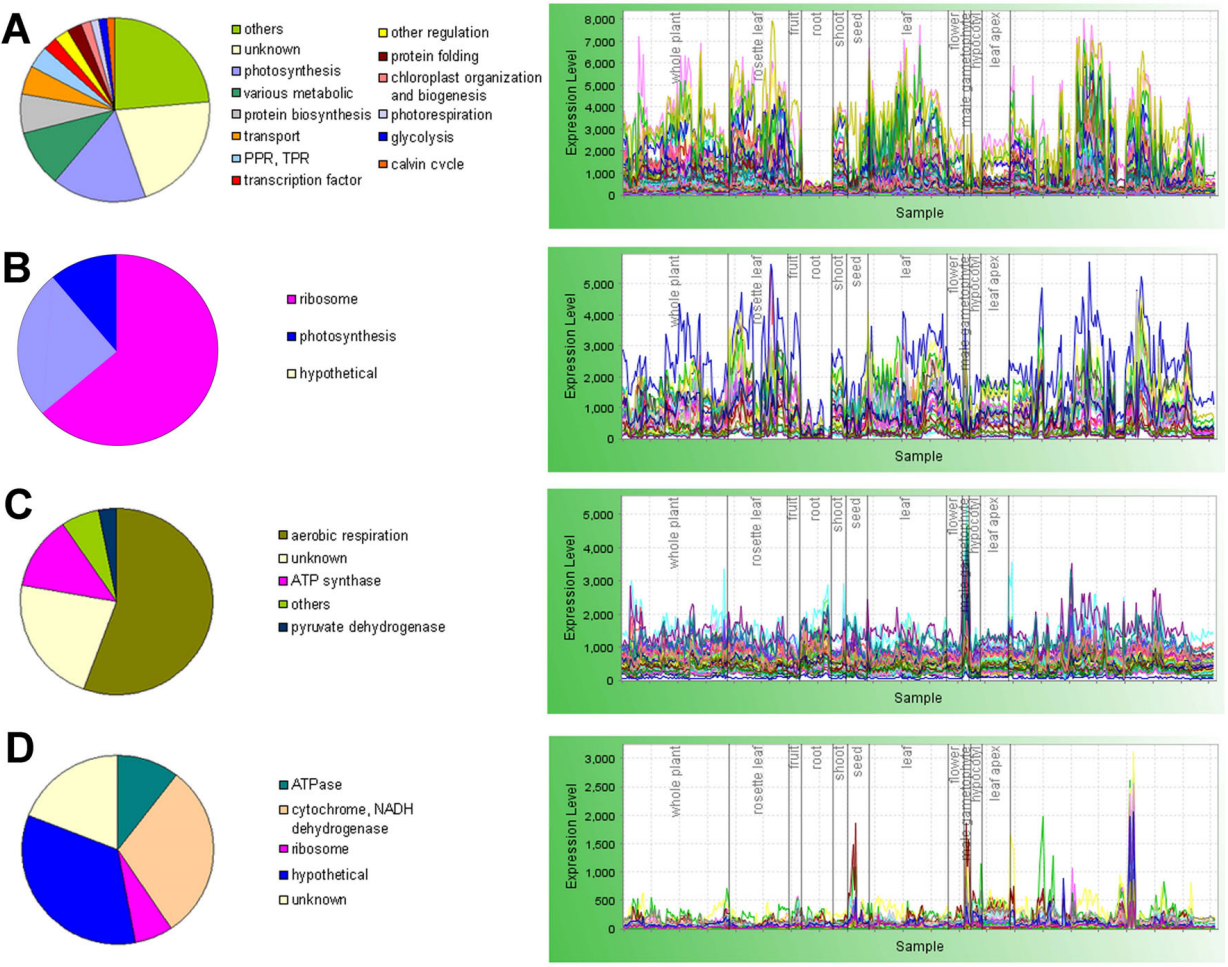
**Regulons with organelle-specific functions and organelle-encoded genes**. Regulon 2, photosynthesis (for clarity, representative expression profiles of 200 randomly chosen genes from this regulon are shown) (**A**); Regulon 49, plastid-encoded genes (**B**); Regulon 29, mitochondrial respiration (**C**); Regulon 34, mitochondrion-encoded genes (**D**). The plots on the right side show expression profiles of the genes in respective regulon (each gene depicted with different color) across the 424 samples in the dataset. The samples have been arranged according to plant tissue. Pie charts are based on manual annotations from published data. RNA profiles plotted using MetaOmGraph [24,124].

Regulon 2, the second biggest cluster, contains 971 mainly nuclear-encoded genes involved in chloroplast biogenesis and photosynthesis (overrepresented GO terms: chloroplast: p-value < 10^-85^, thylakoid: p-value = 1.02 × 10^-28^, photosynthesis: p-value = 1.68 × 10^-15^) (Figure 4A). Nineteen genes are involved in the formation and development of plastid organelle: its biogenesis, organization, fission and relocation. An example of these genes is *thylakoid formation 1 *(PSB29), required for thylakoid membrane organization [62]. Transporters of sodium, calcium and other metals are represented. Two hundreds and eighteen genes in Regulon 2 have a photosynthesis-related activity. Of these, 36 encode enzymes required for synthesis of the photosynthetic apparatus metabolome (porphyrin pigments, tetrahydrofolate, chlorophyll, carotenoids, and other plastidic isoprenoids). Seventy five genes encode plastidic ribosome constituents and related functions. Twenty genes from the Calvin cycle, 16 genes from photorespiration, 14 genes representing a subset of plastidic glycolysis enzymes, and 11 genes involved in starch metabolism are also present, reflecting the coupling of these metabolic activities with the light reactions of photosynthesis. In addition, enzymes for plastidic metabolism of amino acids and nucleotides are represented.

Regulon 2 contains a total of 38 plastid-encoded genes, 27 of which participate in the light reactions of photosynthesis. Such coupling of plastid-encoded and nuclear-encoded genes for the light reactions might be achieved by nuclear-encoded proteins with tetratricopeptide (TPR) or pentatricopeptide (PPR) motifs, which are thought to be transcript-specific regulators of plastome expression [63,64], and sigma factors for plastidic RNA polymerase [65]. Consistent with this concept, 43 genes in Regulon 2 encode proteins with a TPR or PPR domain. One of these, HCF107, has been reported to process the polycistronic chloroplast *psbB-psbT-psbH-petB-petD *operon coding for proteins of the photosystem II and cytochrome b6/f complexes [66]; both *psbH *and *petB *are members of Regulon 2. FLU, another TPR containing protein in Regulon 2, is a negative regulator of chlorophyll synthesis [67]. Five of the six nuclear-encoded sigma factors that modulate the specificity of plastidic RNA polymerase are present in Regulon 2 (SIG1-SIG4 and SIG6 [68]). The exception is the phylogenetically and functionally distinct SIG5, which has been reported to be important for stress response [69,70]; SIG5 is a member of Regulon 15, "plastid stress and circadian rhythm".

Protein import is represented in Regulon 2 by: HCF106, a translocation pathway component that imports proteins into the thylakoid lumen [71]; Tic22 and TOC159, a transit sequence receptor required for import of proteins and essential for chloroplast biogenesis [72]; and CPFTSY and CAO, chloroplast signal-recognition particle receptor proteins [73,74]. Two hundred and three genes in Regulon 2 have no known function. The expression of Regulon 2 is high in light, and in every organ except roots.

### Expression of the plastidic genome is partitioned

The plastid-encoded genes are partitioned across five of the 998 regulons: Regulon 2 (described above), Regulon 176 (8 genes), Regulon 283 (5 genes), Regulon 656 (2 genes) and Regulon 49 (27 genes). In contrast to Regulon 2, with its mixture of nuclear and plastidic genes, the other four regulons contain exclusively plastid-encoded genes: Regulon 49 contains genes for 17 ribosomal proteins and RNA polymerases, seven photosystem-related proteins, and three "hypothetical" proteins (Figure 4B). Interestingly, the operon membership of genes does not necessarily conform to their regulon membership. For example, genes from the tri-cistronic operon, *psaA-psaB-rps14*, each belong to a different cluster (Regulons 49, 2 and 176, respectively), likewise, the genes from the *accD *operon are scattered among three clusters (Regulons 2, 49 and 283).

### Aerobic respiration is a major mitochondrial-related regulon

Only a single regulon of over 20 genes (Regulon 29, 63 genes) contains exclusively nuclear-encoded genes with a predominantly mitochondrial function. Most genes of Regulon 29 are involved in mitochondrial aerobic respiration (Figure 4C). Thirty-nine of these genes encode structural components of the electron transport chain and ATP synthase, six encode TCA cycle enzymes, two code for pyruvate dehydrogenase (one for a cofactor) and twelve are of unknown function. The four remaining genes encode the putative cytosolic galactose kinase GAL1, adenylate kinase, sigma F inhibition-like factor and a mitochondrial dicarboxylate/tricarboxylate carrier. Fifty-one of the 63 proteins encoded by Regulon 29 genes are experimentally demonstrated or predicted to have a mitochondrial localization. The expression is well correlated and highest in pollen.

A large subset of the genes involved in mitochondrial protein synthesis (191 of 869 genes) is contained in Regulon 3, together with genes for protein synthesis in other cell components.

### Mitochondrial genes are integrated with nuclear genes

Seven of the 998 regulons contain an amalgamation of genes from the mitochondrial and nuclear genomes. Although mitochondrial genes predominate in three of these regulons: Regulons 73 (17 out of 18 genes), Regulon 205 (5 out of 7 genes), and Regulon 34 (45 out of 47 genes), no regulon contains exclusively mitochondrial genes. This synchronization of expression of mitochondrial and nuclear genomes is consistent with the recent report that mitochondrial functions require coexpression of genes from both genomes [46].

Of the genes in Regulon 34, 19 represent respiration-related functions (ATPases, cytochrome, NADPH dehydrogenase) (Figure 4D). Most of the other genes are annotated as hypothetical or unknown. Eight are adjacent genes already reported to be co-transcribed (nad3 and rpsL2; rpl5 and cob; nad4L and orf25; atp1 and orf294) [75], however, other genes are from scattered regions of the mitochondrial chromosome. This observed coexpression of genes from different regions of the mitochondrial genome is consistent with experimental evidence that modulation of RNA stability plays a major role in regulation of gene expression in this organelle [75]. The expression of Regulon 34 is generally high and well-correlated, and is upregulated in seeds, male gametophytes, and during starvation.

### Control of nuclear function and information processing

Sixteen regulons are enriched in genes with a genetic information-related function (transcription, translation, replication, DNA metabolism and repair, RNA processing, chromatin assembly, chromatin rearrangement or cell cycle; Table 1).

**Figure 5:**
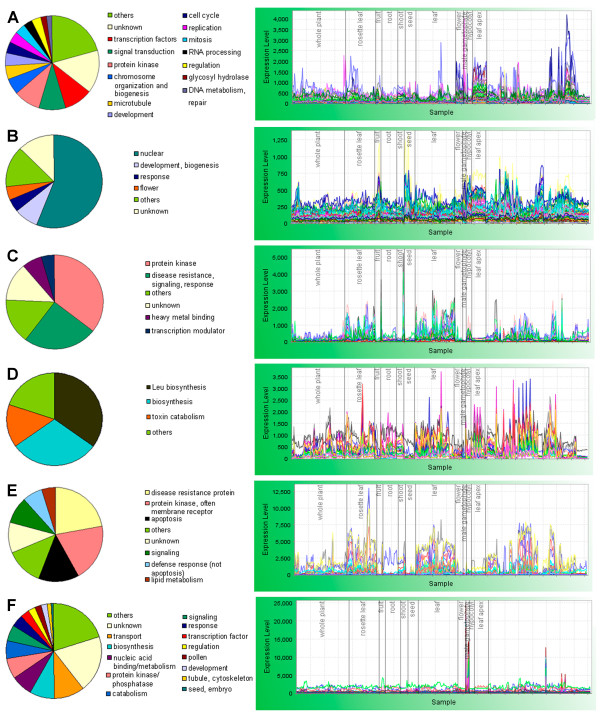
**Regulons with developmental and metabolic functions**. Regulon 4, cell division (for clarity, representative expression profiles of 200 randomly chosen genes are shown) (**A**); Regulon 20, nuclear regulation (**B**); Regulon 35, protein kinases, signaling and defense response (**C**); Regulon 69, glucosinolate biosynthesis (**D**); Regulon 25, defense response (**E**); and Regulon 1, pollen-specific (200 randomly chosen genes) (**F**). Pie charts are based on manual annotation. RNA profiles plotted using MetaOmGraph [24,124].

Regulon 4 contains 495 genes, many with experimentally determined or predicted functions in the cell cycle (Figure 5A). For example, twenty one genes are directly involved in mitosis, including cell division control proteins (CDKB1, CDKB2;1, CDKB2;2, CDC2MsF) and cell division cycle protein HBT, cyclins and other cyclin-dependent proteins. Other nuclear functions represented include gene silencing, regulation of organ development, nuclear transport, RNA processing and histones. Regulatory and signaling genes include 55 transcription factors, 54 protein kinases, 53 signaling-related genes and 18 other regulatory proteins. The expression of Regulon 4 is highest in the leaf apex.

Regulon 20 provides an example of a set of genes involved in nuclear function, which is also associated with a specific developmental process (Figure 5B). Sixty-five out of 94 genes have some kind of nucleic acid-associated activity: transcription factors, splicing factors, chromatin remodeling, histone deacetylases, RNA helicases, DNA repair, RNA processing. However, five genes in this cluster have been implicated in the regulation of flower development: At3g12680, HUA1, is an RNA-binding protein which specifies stamen and carpel identities [76]; At5g04240 (ELF6, early flowering) acts as a repressor of the photoperiod pathway [77]; At2g28290 (SYD) regulates floral homeotic gene expression [78]; At5g17690 (TFL2) controls flowering and floral organ identity by silencing nuclear genes [79]; and At4g32551 (LUG) is a negative regulator of the floral homeotic gene AGAMOUS [80]. No genes reported to be involved in other developmental processes are represented. Rather surprisingly, the expression pattern of this cluster is relatively low and uniform.

### Defense responses

Six regulons contain primarily genes involved in resistance to disease or a pathogen; each is characterized by a set of genes that appears to have a specialized function.

Regulon 35 (45 genes) includes genes involved mostly in a combination of signaling events and responses to pathogens (Figure 5C). Sixteen of the genes encode protein kinases, some experimentally linked to pathogen responses (e.g., CRK5); 10 other genes are involved in disease resistance, response and signaling (cellulase, expansin, six disease resistance proteins, calmodulin- and cyclic nucleotide-binding proteins). Three genes are involved in MAPKKK cascades: MPK3 (a MAP kinase), and MKK1 and MKK2 (MAP kinase kinases). Twenty-three of the encoded proteins have a predicted location in the endomembrane system. At3g56710 (SIB1) is a nuclear protein that modulates transcription in chloroplasts [81] and might coordinate the response of the plastidic genome to the pathogen with the nuclear one. Expression is high in leaves, especially following perturbations by pathogens or during senescence.

Glucosinolates provide a chemical defense against herbivores [82]. Most of the 20 genes in Regulon 69 (Figure 5D) may participate in glucosinolate biosynthesis in chloroplasts. Of the 16 genes annotated with biosynthetic functions, seven have demonstrated or putative involvement in glucosinolate biosynthesis [83], six encode enzymes similar in sequence to those of leucine, homoserine, lysine or choline biosynthesis. Enzymes currently annotated in TAIR by sequence evidence as being involved in leucine biosynthesis might be also active in the glucosinolate pathway, since those pathways have analogous chemical reactions [84,85]. A flavin-containing monooxygenase, an antioxidant involved in glucosinolate production from phenylalanine in rapeseed [86] is also present in the regulon.

Regulon 25 has 70 genes, 19 of which are annotated as disease resistance proteins (Figure 5E). It also contains other genes involved in pathogen response, among them lectin and lectin kinases, genes related to apoptosis, 17 protein kinases, many of them receptors, and eight genes annotated as involved in signaling. Eighteen genes are predicted to be integral membrane genes. The spiky expression of this cluster, highest in leaves, could be considered symptomatic of genes responding to environmental stimuli.

Other regulons predominantly devoted to stress responses include heat shock response (Regulon 37), stress-induced catabolism (Regulon 63), and synthesis of protective compounds derived from shikimate (Regulon 46).

### Tissue-specific regulons

Only sixteen regulons are predominantly expressed in particular reproductive or vegetative structures (flowers- Regulons 21 and 36; flower/fruit – Regulons 12, 43, 44 and 56; pollen – Regulons 1, 8 and 18; leaf apex – Regulon 7; root- Regulons 27 and 59; phloem – Regulon 57; shoot meristem – Regulon 68).

Genes expressed mainly in pollen are grouped in three clusters, Regulons 1, 8, and 18, each having a different expression profile among the samples containing pollen. Regulon 18 is almost exclusively expressed in pollen. Regulon 1, the biggest cluster, is composed of 1623 genes (Figure 5F). It is a very highly correlated cluster and also a very dense one, containing genes with the highest average number of neighbors (154) for each gene. Regulon 1 contains genes involved in the regulation of the pollen development, spermatogenesis and pollen tube growth. Many genes in Regulon 8 have regulatory functions, and many in Regulon 18 are associated with lipid and carbohydrate metabolism and transport. Our clustering confirms the separation of pollen transcriptome into early and late stages of pollen development, observed by Honys and Twell (2004) [87], whose experiment contributes most of the pollen samples in our dataset. Since the number of experiments using pollen tissue in our analysis was small, the temporal resolution of expression of pollen-specific genes may not be high.

In addition to pollen having its own complement of unusually expressed genes, other clusters (Regulons 3, 29, 43, and 52) are highly upregulated in pollen but also in other tissues.

### Coexpression of neighboring genes

We noticed that the genes that are neighbors on a chromosome are often coexpressed. This phenomenon has also been observed by others using different approaches [88-90]. To quantify the extent of the coexpression, we calculated the number of groups of coexpressed neighbors. Coexpressed neighbors are defined as nuclear genes in the same regulon whose Locus IDs differ by at most 20. To eliminate the contribution of tandem gene duplications from this evaluation, arrays of tandem duplicates were removed.

There are 539 groups of coexpressed genes, 1161 genes in total. This value is significantly larger than the number of groups of coexpressed genes from data in which genes are randomly reassigned to regulons (the mean value from random data is 421; Wilcoxon test p-value < 2.2 × 10^-16^; Additional file 1). The groups of coexpressed adjacent genes range in size from two to six genes: 384 of the groups have only two genes and 54 have three genes (Additional file 2).

We were able to take advantage of our assignments of genes to functionally coherent regulons to evaluate whether the phenomenon of coexpressed neighbors is associated with specific regulons or with regulons of particular functions or characteristics. The 539 genes groups of coexpressed neighbors are not members of regulons with any obvious common characteristic or function, nor are they associated with any of the three super-clusters of regulons in the network (photosynthetic functions, information processing, and stress responses). Also, the coexpressed neighboring genes are not enriched in any GO term. Thus, coexpressed neighbors don't seem to be associated with a particular function, either with respect to gene annotation or participation in particular regulons.

**Figure 6:**
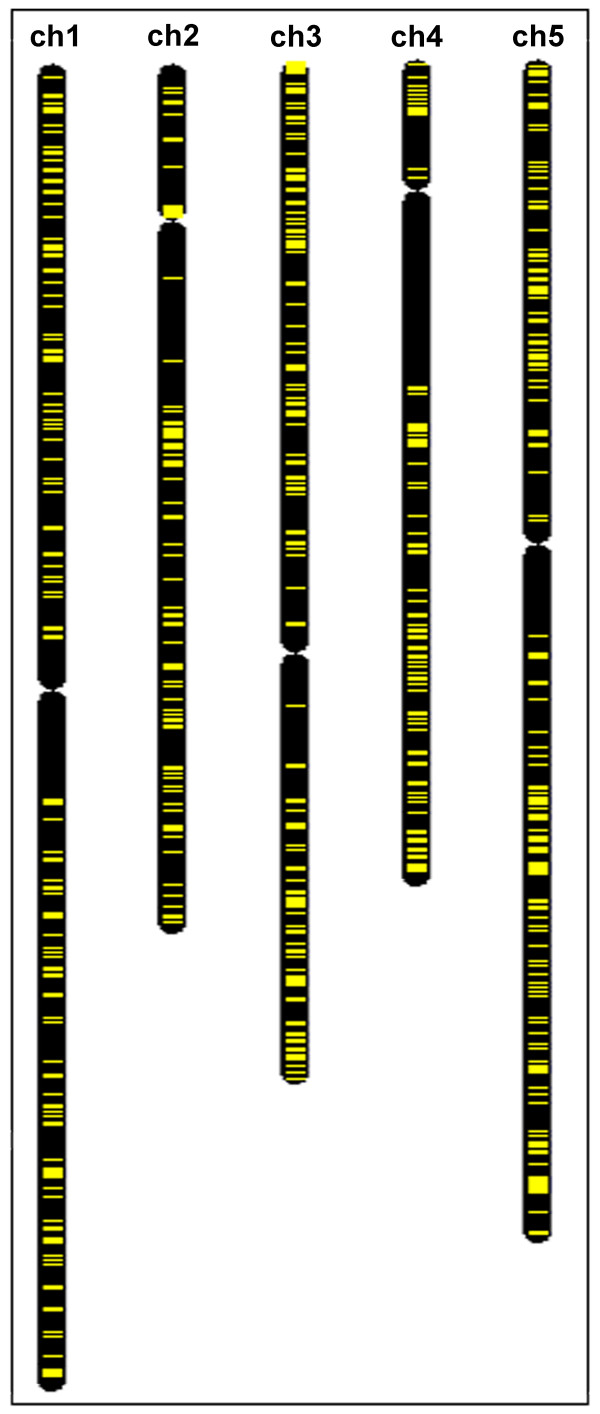
**Coexpressed neighboring genes are absent from the region of long arm of chromosome 4**. Distribution of the coexpressed neighboring genes (marked in yellow) on five Arabidopsis chromosomes (visualized in Chromosome Map Tool, [126,115]). Domains of coexpressed neighbors are absent from large part of the long arm of chromosome 4, adjacent to the pericentromeric region, and very rare in the analogous area of chromosome 2.

Interestingly, the distribution of groups of coexpressed neighbors on the chromosomes is not uniform. Domains of coexpressed neighbors are absent from large part of the long arm of chromosome 4, adjacent to the pericentromeric region, and very rare in the analogous area of chromosome 2 (Figure 6).

### Genes of unknown function

The unknown genes that co-cluster with genes of known function might be hypothesized to share that function with characterized genes. Recently, Horan et al. [16] used the clustering of public expression data to assign function to genes coding for proteins of unknown function (PUF), defined as genes with a GO term GO:0003674 (unknown molecular function). This way authors have proposed a function to 277–1541 PUFs, depending on the significance threshold. Of the 277 PUFs assigned to clusters with the highest confidence in Horan et al., 216 are present in our 998 regulons. The highest number of those 216 PUFs belong to Regulon 2 (photosynthesis, 94 PUFs), 19 are present in Regulon 3 (protein synthesis) and 12 in Regulon 4 (mitosis).

In our analysis, the total of 2896 PUFs have been assigned to 148 larger regulons with at least 10 members. In 69 largest regulons there are 2584 PUFs, 1768 PUFs in only 10 largest regulons. PUFs are approximately proportionally distributed and consist about 30% of regulons. Thus, the functions most often assigned to PUFs are that of Regulon 1 (pollen-specific, 457 PUFs), Regulon 2 (photosynthesis, 327 PUFs) and Regulon 3 (protein synthesis, 213 PUFs). The exceptions are regulons 49 (plastidic genes), 53 (fatty acid biosynthesis), and 69 (leucine/glucosinolates biosynthesis) from which PUFs are entirely absent.

### Genes of extremes

**Figure 7:**
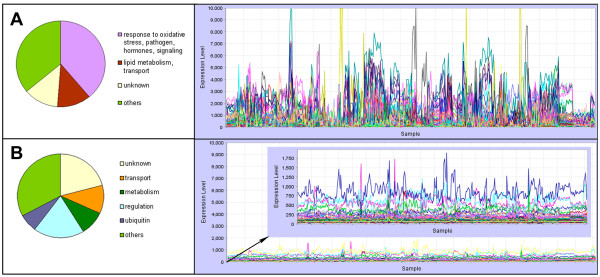
**Functional assignments and expression profiles of genes with the most and the least variable expression across multiple conditions**. **(A) **100 genes with the most variable expression (highest standard deviation of logE). **(B) **100 genes with the most steady expression (lowest standard deviation of logE). The scale along Y axis (expression values) is the same for both plots to facilitate comparison of the expression profiles between them. Inlet shows a version of plot B with zoomed scale of expression values.

We identified the genes with highly varied expression, little variation in expression, as well as those genes that had sub- or super- mean levels of expression, from the expression profiles of the 22,746 probes in the Arabidopsis ATH1 chip using the same 963-chips dataset. To evaluate whether the genes with extremes in expression patterns have any particular characteristics, the functions of the 100 genes with the most varied expression, the steadiest expression and also those with the highest and lowest expression were assigned to functional classes based on TAIR and GO annotations and manual curation (Figure 7 and Additional file 3).

Genes with greatly shifting expression patterns across a wide variety of conditions might be considered candidates for responses of the plant to internal and/or external signals. We defined the genes with the most dramatically shifting expression profiles as 100 genes with the highest standard deviation of logged expression value. Indeed, 39 of these 100 genes had annotation suggesting their involvement in signaling (reaction to stimuli, p-value 0.0765; response to oxidative stress, pathogens or hormones) (Figure 7A and Additional file 4). Only a single metabolic function is included: 12 genes had a function related to lipid metabolism, lipid transport, or lipid degradation, possibly reflecting fluctuating requirements for energy and membrane synthesis. The endomembrane system is highly overrepresented (p-value 9.2 × 10^14^).

Genes with the steadiest expression were defined as the 100 genes with the lowest standard deviation of logged expression value. Twenty-six of these genes are relatively highly expressed, having a mean level of expression greater than 100. The group of most evenly expressed genes includes a conglomerate of metabolic, regulatory, and transport functions (Figure 7B and Additional file 5). There is a high proportion of "unknown" genes in this group (21%); possibly the steady level of expression of these genes would make it more difficult to ascertain their function.

A related result was obtained with a different approach, focused on responses in stress-related microarray experiments, applied by Walther et al. [91]. The authors found that genes annotated as responding to the various stimuli were differentially expressed in the highest number of experiments, while those with unknown or house-keeping functions had the smallest breadth of response.

Genes with the highest expression (defined as genes with the highest mean of expression values across all samples) include photosynthesis-related genes (p-value 9.28 × 10^-26^), and structural constituents of ribosomes (p-value 2.26 × 10^-09^) and other genes for protein biosynthesis and modification. Together, these functions constitute 58% of annotations of genes expressed at the highest level (Additional file 3 and Additional file 6).

The majority of the genes expressed at the lowest levels (defined as the 100 genes with the lowest mean of expression values across all samples) are predominantly hypothetical genes, transposons and pseudogenes (Additional file 3 and Additional file 7); these are classes of genes for which no or little expression might be expected. Twenty-three of the genes in low expression group have functional annotations, including nucleic acid-binding genes and disease resistance genes. Because the expression for genes in this group does not exceed 40 for any chip, a significant component of the signal may be an artifact (e.g., due to signal processing or normalization).

### Negatively correlated pairs of genes

These analyses also yield information about which gene pairs are negatively correlated. Wei et al. [51] noted that within the subset of 1330 annotated metabolic genes, there are few negative correlations. Interestingly, this paucity of negative correlations is also true when the genes other than metabolic (e.g., regulatory and unknown) are examined. In our dataset, negative correlations are far less abundant than positive ones; the highest negative value in the dataset is -0.73. This value for negative correlation may be an underestimate, since Pearson correlation coefficient measures the amount of linear relationship, while negatively correlated genes appear to have reciprocal relationships. Interestingly, in six out of the eight most negatively correlated pairs of genes, one or both genes are implicated in a regulatory function.

## Discussion

The picture of Arabidopsis emerging from this study is that of a plant mainly occupied with gathering energy, reproduction and defense from a hostile environment. Genes involved in photosynthesis and photosynthesis-related metabolic processes are highly expressed, and form the second largest regulon. Several other regulons appear to mediate chloroplast development and aerobic respiration. Developmental programs associated with reproduction account for the function of ten clusters and include pollen, flower, fruit, root, and embryo. Maturing pollen-specific genes form the largest and the most highly correlated regulon, comprised of the 1623 genes. Response and signaling programs are diverse and abundant, reflecting the need in the realization of the genetic program for elasticity in response to changing conditions. Thirteen of the 69 regulons with at least 20 gene members appear to mediate plant responses to external or internal stimuli. Each of these response-related regulons contains a mixture of molecular functions: receptors, kinases, hormone signaling, and metabolic genes required for defense (for example, enzymes for degradation of a pathogen cell wall). Response-related genes are also among the most variable with respect to expression level.

### Comparison with Graphical Gaussian Model

The graphical Gaussian model (GGM network) presented by Ma et al. [54] is a network which contains only links that signify direct dependencies between genes. Our purpose was a bit different – we wanted to see all the genes whose response in expression in various conditions is similar, irrelevant if the effect on them is direct or not, in order to learn the organization of the plant cell transcriptome. Similarity of expression between connected genes is precisely the meaning of the link in our coexpression network. Thus, with no restrictions for the associations to be direct, our network is much larger, containing 13,456 genes connected by almost 1.5 million links. A method for exhaustive comparison between these two networks would be problematic, because only 4749 genes are shared between them, and because we analyze our network by partitioning it globally into densely interconnected subnetworks, while Ma and coworkers query the local neighborhoods of the selected seed nodes.

However, generally, we observe that genes from our genetic information-related regulons are largely missing from the GGM network (over 90% of genes from Regulons 3, 11, 20, 26, 32, 38, 40–42, 50, 60–61, 64 and 66 are absent). On the other hand, genes from metabolism-related and organelle-encoded regulons are very well represented in GGM (over 90% of genes from Regulons 21, 22, 23, 30, 34, 37, 44, 46, 49, 56, 59, 65, 70 are present).

A network formed by genes for cellulose biosynthesis is one of the most characterized in Arabidopsis [44,45]. Both in our network and in the GGM network, the cellulose biosynthesis genes were partitioned into those specific to primary and secondary cell wall biosynthesis. However, our regulon representing secondary cell wall (Regulon 22) is more comprehensive, containing 81 genes, compared to 41 in Persson et al. [44], and 64 in GGM; 34 of those 81 genes have an annotation consistent with cell wall biosynthesis, including laccases and microtubule-associated proteins linked with this process [43,92]. The three primary cell wall biosynthesis genes (CESA1, CESA3 and CESA6) are part of the 12-gene regulon (Regulon 121) which also includes drought and cold-responsive genes. Drought and cell wall biosynthesis have been linked before, as the modulation of the rate of the cellulose biosynthesis provides a mechanism to cope with dehydration [93,94]. Likewise, the 11 genes designated as "proteasome complex" in GGM are part of a bigger module in our network: 9 out of these 11 genes are present in Regulon 45, along with 17 other genes encoding proteasome subunits. On the other hand, most of the genes that form the local subnetwork related to cytokinin-mediated signaling in GGM are not present in our network. Some of the subnetworks identified in GGM are further partitioned in our data into more specialized regulons. For example, 9 out of 20 chromatin-related genes belong to Regulon 4 (mitosis), while 3 belong to Regulon 84 (regulation of flower development). Similarly, flavonoid biosynthesis genes form Regulon 93 (lignin/lignan biosynthesis; 12 of the 14 genes have confirmed or putative functions as catalyzing reactions from synthesis of shikimate through generation of lignin/an monomers) and Regulon 146 (flavonoid/rhamnoflavanoid synthesis; 9 of the 10 genes have confirmed or putative functions catalyzing reactions from chalcone synthase to rhamnose transferase).

### Implications for metabolic pathways modelling

Our analysis reflects a clear dichotomy between the metabolic pathways of textbooks and the Arabidopsis transcriptional network. By identifying genes that are co-regulated with particular metabolic fluxes, and experimentally evaluating the effect of these genes on these fluxes, the fundamental mechanisms underlying regulation of metabolism can be better understood. One fundamental question is the extent of the integration of regulatory, metabolic and structural genes within a regulon. Genes for a number of metabolic pathways have been shown to be coexpressed (eg., [42,51,46]), and pathway genes from the AraCyc metabolic pathway database also have coexpressed regulatory genes [51]. In contrast, in a clustering of 3292 nuclear-encoded genes for plastid-localized proteins, Biehl et al. [50] identified regulons that, with the exception of photosynthesis and plastid protein biosynthesis, were composed of genes with diverse pathway associations. The global clustering described herein places these observations in the context of the near-whole Arabidopsis transcriptome. Although our analysis of a correlation among the 22,746 probes on the ATH1 chip clearly reflects the co-occurrence of enzymes from metabolic pathways in regulons, the aggregation of single metabolic pathways into distinct regulons is surprisingly scarce. Exclusively (or nearly exclusively) metabolic regulons are: fatty acid synthesis (Regulon 53), aerobic respiration (Regulon 29), glucosinolate biosynthesis (Regulon 69), lignin/lignan biosynthesis (Regulon 93), flavonoid synthesis (Regulon 146), and protein biosynthesis (Regulon 3).

One possible explanation of why a given metabolic pathway may not form stand-alone regulons is that it is part of a biological program encompassing several concurrent and mutually dependent processes. Thus, the clusters of enzymes found to be coexpressed by Gachon et al. [42] in their analysis of secondary metabolite pathway genes also are identified in our analyses, however, most are contained within regulons that combine regulatory functions with structural genes and related metabolic steps. For example, shikimate biosynthesis genes are part of Regulon 46, which contains genes involved in defense responses, including biosynthesis of protective compounds. Similarly, genes from plastidic glycolysis and the Calvin cycle cluster with other genes active in the chloroplast during photosynthesis (Regulon 2).

In a few cases, such as sucrose biosynthesis, or glycolysis, metabolic pathways are dispersed across multiple regulons. Metabolic pathway might not form a discrete regulon because of participating enzymes having multiple metabolic functions, e.g., enzymes of cytosolic glycolysis are utilized for respiration and for anapleurotic reactions [95]. A second possibility is that enzymes from the pathway may be under translational or post-translational regulation. Finally, gene families may confound co-expression patterns, as multiple genes in a family may contribute under different conditions to a single enzyme.

### Regulation of expression of organellar genomes

Our results agree with a body of experimental literature (reviewed in [96]), which indicates that the expression of the 130 genes of the plastidic genome is not uniform and is apparently finely tuned by multiple levels of regulation. Genes from the plastidic genome fall into five regulons of varied functions: Regulon 2 (predominantly encoding proteins of photosynthesis and related metabolism); Regulon 176 (predominantly encoding proteins of PSI); Regulon 49 (mainly ribosomal proteins); and Regulons 283 and 656 (two small clusters of mixed function). In general, all genes from a given operon are in the same regulon, suggesting that transcriptional regulation of the plastid genome is a major determinant of transcript accumulation. However, in some cases, genes from a single operon are dispersed across multiple regulons. Thus, this pattern of regulon organization is likely a reflection of two processes: accumulation of subsets of transcripts driven by distinct PEP/NEP promoter combinations, and modifications of transcript levels due to alternative RNA processing, mediated by nuclear processing factors such as PPR proteins.

Individual regulons may contain many levels of regulation, as exemplified by Regulon 2, which appears to participate in the building and function of the photosynthetic machinery and related metabolic processes. The two genomes that cooperate to meet this goal, nuclear, encoding the majority of the plastid-localized proteins, and plastidic, [97] both are represented in Regulon 2. Coordination of these two genomes requires anterograde (nucleus to plastid) signaling mechanisms (e.g., regulation of transcription of nuclear-encoded plastid proteins, import of proteins into plastids, plastid genome transcription rate and specificity, photosynthetic complex assembly, or plastid development by nuclear-encoded factors) as well as retrograde (plastid to nucleus) signaling mechanisms (e.g., via redox state, chlorophyll synthesis intermediates, sugar or singlet oxygen signaling) [98-101]. Since genes experimentally identified as participating in many aspects of anterograde and retrograde signaling are coexpressed in Regulon 2, we infer that a complex network may modulate photosynthesis-related activities in this regulon.

Interestingly, of the 998 regulons, none contains genes from both organellar genomes (mitochondrion and plastid). This suggests that coordination between these organelles may not be achieved by transcriptional co-regulation. Furthermore, that all plastid-encoded genes, except those mobilized in Regulon 2, are grouped in regulons by themselves (not with any nuclear gene) underscores the independence this organelle has maintained hundreds of millions years after endosymbiosis.

### Relationships among the regulons

The grouping of regulons with information-related, stress response, and plastid-related functions in dense regions of the transcriptional network indicates that genes in these dense regions may participate in multiple related genetic programs that under some subsets of conditions are coexpressed. Other studies have shown that stress response genes are not usually specific and react to several types of stress [102]. In contrast, some regulons are relatively isolated from the network, most notably, Regulon 53, fatty acid synthesis, and Regulon 34, containing mitochondrial genes. Isolated regulons might contain genes that are committed to a discrete process that is carried out in relative independence from other cellular functions.

### Genes not included in the network

During the data preprocessing, we have filtered out genes with low expression and with no similarity to other genes (Fig. 1). The genes that code for proteins located in mitochondrion or endomembrane, or involved in apoptosis or regulation of transcription, were among those with the least expression. Many genes for membrane-located proteins, including transporters, had expression profiles unlike any other in the genome. Both these low-expressed and unique-profile classes of genes were not used for network construction. Although many of low-expressed genes are hypothetical and might not be transcribed (76 out of 100 genes with lowest expression in our data were not expressed in the experimental evaluation of the Arabidopsis expression activity by whole genome tiling array by Yamada et al. [103]), some of the hypothetical genes might be active, but in very specific cell types or temporal conditions and thus their expression or ESTs have never been detected. Several well studied genes, for example regulators of flowering CONSTANS and FRI, or myb-type transcription factor CPC, responsible for differentiation of the epidermal cells, are expressed at a very low level and were filtered out of our analysis. Furthermore, genes with flat expression profiles might also have little representation in the network; only 14 out of the 100 genes with most steady expression profiles were incorporated into a cluster, while 68 were filtered out due to low similarity to any other gene. The profiles of regulatory genes may be flat because the activity of their products is often modulated by translational or post-transcriptional modification – addition of a phosphate group, induced conformation change, binding a cofactor or other subunit. Because regulatory genes are among the most comprehensively studied and often helped to guide the designation of cluster function, their absence in clusters hinders the identification of developmental programs in our clustered data.

### Coexpression of neighboring genes

The coexpression of neighboring genes identified in this analysis is higher than expected by chance and cannot be explained by coexpression of tandem duplicates. This result is consistent with several reports, each applying different definitions of coexpressed neighbors and using various methodologies to identify such groups [88-90]. The small sizes of domains of coexpressed neighbors in our data also agree with reports that coexpression is a short distance effect in Arabidopsis. Several hypotheses have been raised to account for observed local coexpression, such as that the coexpressed neighbors reside in chromatin domains with open conformation [104,105,90], have shared regulatory cis-elements [106], or are organized into eukaryotic operons [107-109]. Our analysis indicated an unusual distribution of coexpressed neighbors on chromosomes along with an absence of overrepresentation of any biological function in co-expressed neighbors; both these observations are in accordance with the hypothesis that chromatin structure is the key player in the local coexpression effect.

### Negative correlations

Pairs of negatively correlated genes might merely reflect disjoint sets of conditions in which the two genes are active; alternatively, they might indicate a regulatory relationship. The fact that most of the stronger negative correlations observed in this analysis include regulatory proteins is consistent with a possible biological importance of negative correlations. In agreement with this interpretation, our analysis identifies negative regulations that have already been established experimentally. For example, At2g23430 (ICK1), cyclin-dependent kinase inhibitor protein, functions as a negative regulator of cell division and interacts with CYCD3;1 [110]; in our analysis, ICK1 is negatively correlated with CYCD3;1 (Pearson's R = 0.43). ICK1 also negatively correlates with the cyclin-dependent protein kinases CYC2b and CYCA2-similar, cell division control protein CDKB2;1 and other mitosis-related genes (Pearson's R = 0.52 to 0.56). In a second example, At1g75950, SKP1, a negative regulator of DNA recombination [111], is most negatively correlated with DNA polymerase and tubulin-related genes (R = -0.5 to -0.4).

Whether a given negative *correlation *translates to a negative *regulation *must be experimentally evaluated. An example of a pair of genes that are highly negatively correlated (R = -0.73) is At1g06650 (2-oxoglutarate-dependent dioxygenase similar to tomato ethylene synthesis regulatory protein E8) and At5g23430 (transducin family protein with nucleotide binding WD-40 repeat). Another example is At3g11910 (DNA binding ubiquitin-specific protease) and At4g12800 (photosystem I reaction center subunit XI) (R = -0.73). A testable hypothesis of the later correlation is that this ubiquitin-specific protease may play a role in the turnover of the photosystem I reaction center protein associated with photodamage.

### Availability of the data

The regulons data have been incorporated in MetaOmGraph software for visualizing and analysis of large datasets within the MetNet Platform [112]. Regulons can be downloaded as the gene sets. The user can view expression profiles of the regulons across all experiments, or in a subset of experiments, examine the gene contents of the regulons and calculate the values for the absolute and signed versions of Pearson and Spearman correlation between the genes.

## Conclusion

This analysis yields insight on the organization of plant transcriptome into concerted processes. The network provides an initial glimpse of the interactions among regulons in a broad biological context. Moreover, this study has the potential of assigning function to un-annotated and partially annotated genes; nearly 3000 genes of "unknown" molecular function have been assigned to a regulon. As such, it provides new, experimentally-testable hypotheses about the functions of genes. Further analysis of functionally coherent regulons will enable refining the existing models of metabolic regulation, developmental and response programs, and intergenomic communication.

## Methods

### Transcriptome data

Arabidopsis expression data for 963 Affymetrix ATH1 chips with 22,746 probes were obtained from Nottingham Arabidopsis Stock Centre microarray database [19,18] and PLEXdb [23,56]). The data represent 70 experiments, including development, stress, mutant, and other studies. All chips obtained from NASC database were already individually scaled with MAS 5.0 algorithm (Affymetrix) to the common mean = 100, excluding top and bottom 2% signal intensities. The data in PLEXdb are MAS5-normalized with mean expression of chips set to 500. To make the data from these two databases comparable, we scaled the data from PLEXdb database to set the chip mean to 100. The reproducibility of experiments was assessed by visual inspection of scatter plots and by applying a threshold of R^2 ^> 0.86. Chips with poor biological replicates were discarded. The remaining biological replicates were averaged to yield 424 samples. The data was subsequently normalized to the same range by a median absolute deviation (MAD)-based scale normalization method described by Yang et al. [113]. MAD-based scale normalization was chosen instead of the quantile normalization methods in order to minimize the interference with the data. Expression values *x*_*ij *_on microarray chip *j *were multiplied by the factor $\frac{C}{MAD_{j}}$, where *MAD *is defined by

*MAD*_*j *_= median_*i*_{|*x*_*ij*_-median_*i*_(*x*_*ij*_)|}

and the constant C is an arithmetic mean of *MAD*

$$C=\frac{\sum_{j=1}^{n}MAD_{j}}{n}$$

Because our dataset is rather large, consisting of 424 datapoints, and the distribution is generally assumed to be approximately normal when the number of datapoints exceeds 100, the data has not been log-transformed.

The normalized data, together with its metadata is available online [24]. The 70 experiments in the dataset are also listed in the Additional file 8. ATH1 probe set-to-Locus ID mapping was obtained from TAIR [114,115].

All computations in this work, except for graph clustering, were performed in R software [116].

### Generating the network of coexpressed genes

Because low expression values are not reliable and might introduce noise in the dataset, the 4551 of the 22,746 gene probes on the Arabidopsis ATH1 chip, whose expression was < 100 (the mean of the gene expression values on the chip) in all samples were filtered out. A Pearson correlation matrix was calculated for the remaining 18,195 genes. Of these, only the 14,564 genes that were correlated above the Pearson correlation threshold of 0.7 with any other gene were retained for further analysis. The matrix was transformed into a binary matrix by replacing the values of all correlations > 0.7 by 1, and assigning the others as 0. The resulting binary matrix induced the adjacency matrix of the coexpression network, in which genes form the nodes and two genes are connected by an edge if they are correlated above 0.7.

This Pearson correlation criterion of 0.7 was developed on the basis of our previous results of coexpression analysis of three metabolic pathways (fatty acid biosynthesis, leucine catabolism and starch metabolism) that was performed on the same expression dataset [117]. In this previous work we observed the emergence of specific (i.e. within-pathway) links from the background noise (intra-pathway links) with the increase of the Pearson correlation threshold from 0.5 to 0.7.

### Clustering the coexpression network

Connected components were identified in the network (*connectedComp *function in R software), yielding one giant connected component with 14,368 nodes and 77 smaller components, ranging from 2 to 8 nodes. Because we aimed to find strongly inter-connected clusters, genes connected only by a single edge were removed from the biggest connected component, and the resulting network composed of 13,456 genes and nearly 1.5 million edges was clustered by Markov chain graph clustering algorithm [114,58] with the *inflation *parameter set at 1.8. An array of the inflation parameters has been assessed based on the degree of integrity of three metabolic pathways (fatty acid biosynthesis, leucine catabolism and starch metabolism) in the corresponding clustering results. The inflation value of 1.8 was chosen because it resulted in the best correspondence between the clusters and the sets of genes from the same pathway. Nine hundred and ninety eight clusters were produced (see Additional file 9 online for the complete assignment of genes to the regulons); these were analyzed together with smaller connected components from the previous step. The network was visualized (see Figure 3) using the GraphExplore tool [118] in a simplified representation, in which the nodes represent clusters (regulons) and an edge joins two clusters if there exists an edge between any pair of genes belonging to these two clusters in the underlying network. This criterion was chosen because of the large differences among the number of between-cluster links.

### Clustering significance

The significance of our clustering results was assessed by comparison of the overrepresentation of GO terms in the 148 regulons identified from the experimental microarray data with 10 or more genes, to the overrepresentation of GO terms of 100 sets of 148 randomly-obtained clusters. Each of these random sets was obtained by permuting the gene IDs so that the permuted cluster sizes were the same as the real ones, but genes assignment to the clusters changed. Each clustering *i *was assigned a score *S*_*i*_. For this value, the best p-value *p*_*min *_for overrepresentation of any GO term was recorded for each cluster and averaged over all clusters.

$$S_{i}=\frac{\sum_{j=1}^{n}p_{\min j}}{n}$$

where *n *denotes the number of clusters (n = 148).

Distribution of S values for GO Molecular Function, Biological Process, and Cell Compartment for randomly-assigned groups were compared to the respective values for the real clustering. In each case, the real value scored significantly better than any of the random ones (Wilcoxon test p-value < 2.2 × 10^-16^).

To compare the overrepresentation of GO terms in clusters obtained by MCL clustering with that of clustering produced by k-means algorithm, the *kmeans *function in the *stats *package in R was used on the original expression dataset, with the same number of clusters (998) as a parameter. Only 471 k-means clusters with at least 10 members were used in the comparison. The adjusted *rand *indexes (*classAgreement *function in *e1071 *package in R [119]) were calculated to quantify agreement between gene assignments to regulons by MCL and k-means clustering algorithms.

The Z-scores for mutual information between clusterings and GO terms were calculated according to Steuer et al. [120]

$$S=\frac{MI{\left(C,A\right)}_{real}-mean(MI{\left(C,A\right)}_{random})}{\sigma_{random}}$$

where MI(C, A) denotes the mutual information between the clustering and the GO terms attributes, and σ_*random *_denotes the standard deviation of the MI(C, A) in the randomized data. MI(C, A) was calculated from the contingency table that contained the counts of 333 GO terms for genes in 148 larger clusters with at least 10 genes (305 GO terms and 471 clusters, respectively, for k-means clustering). GO terms included all terms associated with any gene in the 148 (471) larger clusters, after removing rare terms (associated with less than 10 genes in the clustering) and one of each pair of redundant GO terms (that differ in characterization of less than 10 genes). Random data was obtained as before, by randomizing assignments of genes to clusters while preserving the sizes of the clusters.

The mapping of Arabidopsis genes to GO terms was obtained from TAIR [121]. The modified *GoHyperGall *function in R module *Bioconductor *was used to obtain batch results of overrepresentation of GO terms.

### Analysis of functional coherence

The coherence of functionality of the genes within each clusters of at least twenty genes was assessed by a combination of automatic analysis of overrepresentation of GO terms [122,123] and manual inspection of function and expression, using the published literature and tools (MetaOmGraph, AtGeneSearch) in the MetNet Platform [112,124]. The RNA profiles were visualized and plotted in MetaOmGraph.

### Coexpression analysis of the neighboring genes

Coexpressed neighbors were defined as those nuclear-encoded genes in the same regulon, whose Locus IDs differ by at most 20. Arabidopsis Locus IDs are in the form ***At****x****g****yyyyy*, where *x *denotes the chromosome, and the number *yyyyy *reflects the order of genes on the chromosome. Only one gene from each array of tandem replicates was used in the calculation of the number of genes in groups of coexpressed neighbors. Tandem replicates were identified with AGI software [125] using BLASTP with a threshold of *E *< 10^-20 ^and allowing for one unrelated gene among cluster members.

For identification of groups of coexpressed neighbors in randomized datasets, nuclear-encoded genes were reassigned to regulons randomly, and the number of coexpressed neighbors was determined using the same criteria as for the experimental data. The mean number of groups of coexpressed neighbors in 100 reshuffled datasets was 421, compared to 539 in the real dataset. Overrepresentation of GO terms in the genes that form the groups of coexpressed neighbors was evaluated in GOstat web tool [123].

## Abbreviations

GGM: Graphical Gaussian Model; GO: Gene Ontology; MCL: Markov cluster algorithm; PEP: plastid-encoded RNA polymerase; NEP: nuclear-encoded RNA polymerase.

## Authors' contributions

WIM designed the study and conducted the analyses, WIM and ESW wrote the manuscript. Both authors read and approved the final manuscript.

## Supplementary Material

Additional file 1 **Distribution of the numbers of groups of coexpressed neighboring genes in 100 randomized datasets**. The nuclear-encoded genes were randomly reassigned to the regulons. Groups of coexpressed neighbors were counted in the same way as in the real dataset. The mean number of coexpressed groups in 100 randomized datasets was 421.4, compared to 539 in the real dataset Click here for file

Additional file 2 Distribution of the sizes of groups of coexpressed neighboring genes in experimental and randomized data Click here for file

Additional file 3 Functional assignments and expression profiles of the 100 genes with (A) the highest expression (maximum mean), and (B) the lowest expression (minimum mean). Click here for file

Additional file 4 Genes with the most changing expression. Click here for file

Additional file 5 Genes with the most steady expression. Click here for file

Additional file 6 Genes with the highest expression. Click here for file

Additional file 7 Genes with the lowest expression. Click here for file

Additional file 8 Experimental metadata. Click here for file

Additional file 9 Assignment of genes to 998 regulons. Click here for file
