# Supplementary figures and images for: Regulon organization of Arabidopsis
Source: BMC Plant Biol. 2008 Sep 30;8:99. doi: 10.1186/1471-2229-8-99 (PMC2567982; doi:10.1186/1471-2229-8-99)

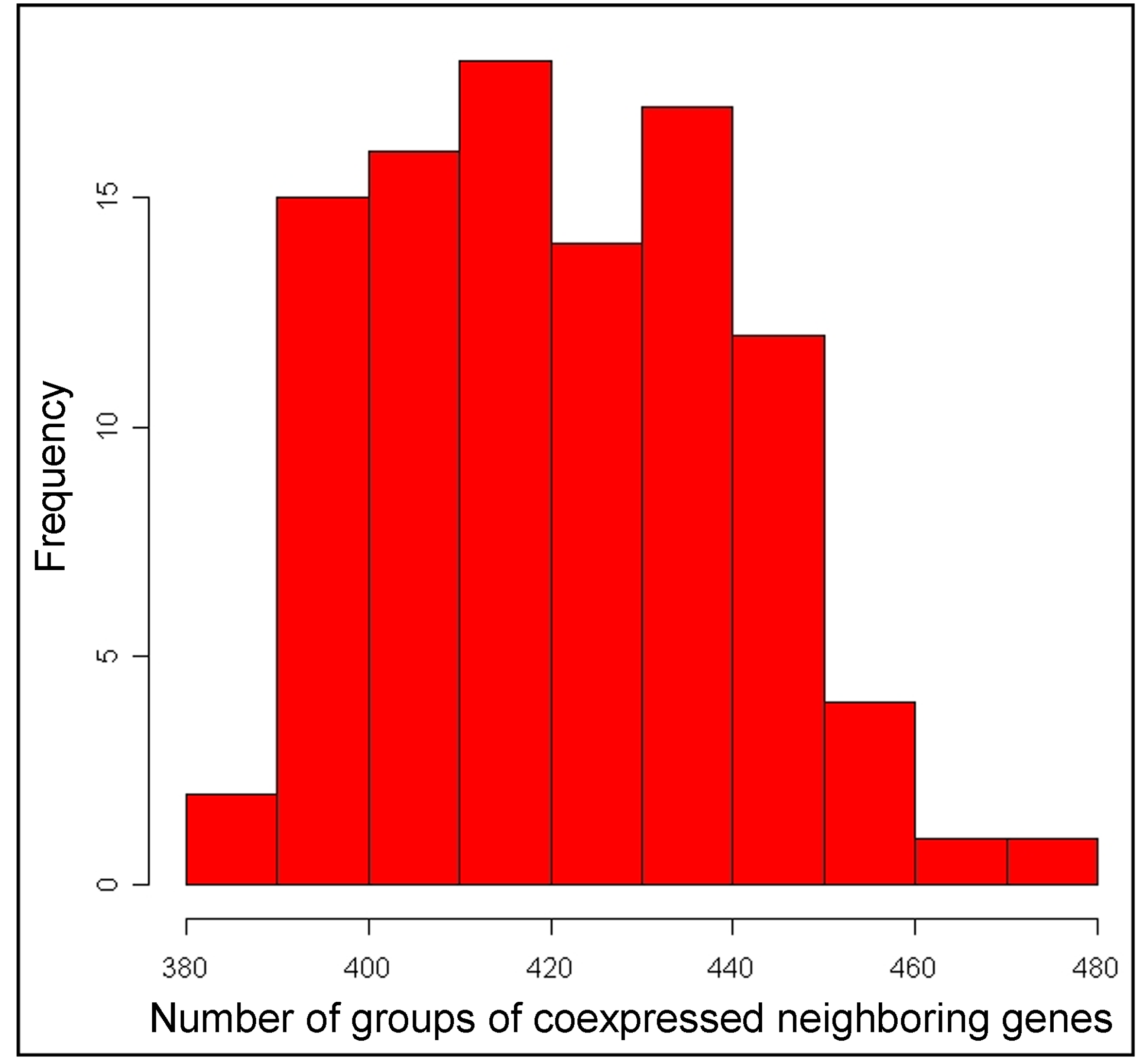

Supplement: Additional file 1 — Distribution of the numbers of groups of coexpressed neighboring genes in 100 randomized datasets. The nuclear-encoded genes were randomly reassigned to the regulons. Groups of coexpressed neighbors were counted in the same way as in the real dataset. The mean number of coexpressed groups in 100 randomized datasets was 421.4, compared to 539 in the real dataset [file 1471-2229-8-99-S1.tiff]

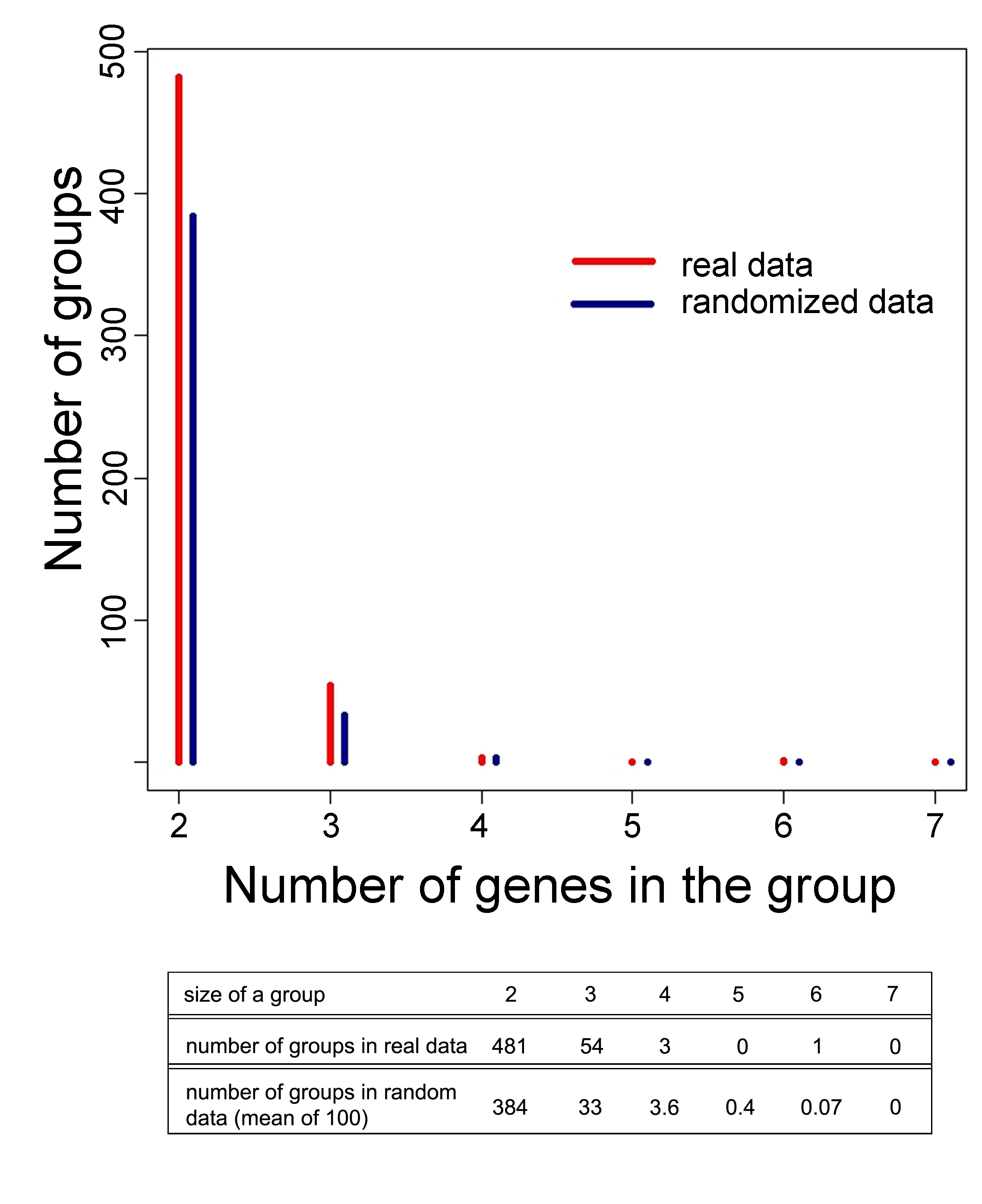

Supplement: Additional file 2 — Distribution of the sizes of groups of coexpressed neighboring genes in experimental and randomized data [file 1471-2229-8-99-S2.tiff]

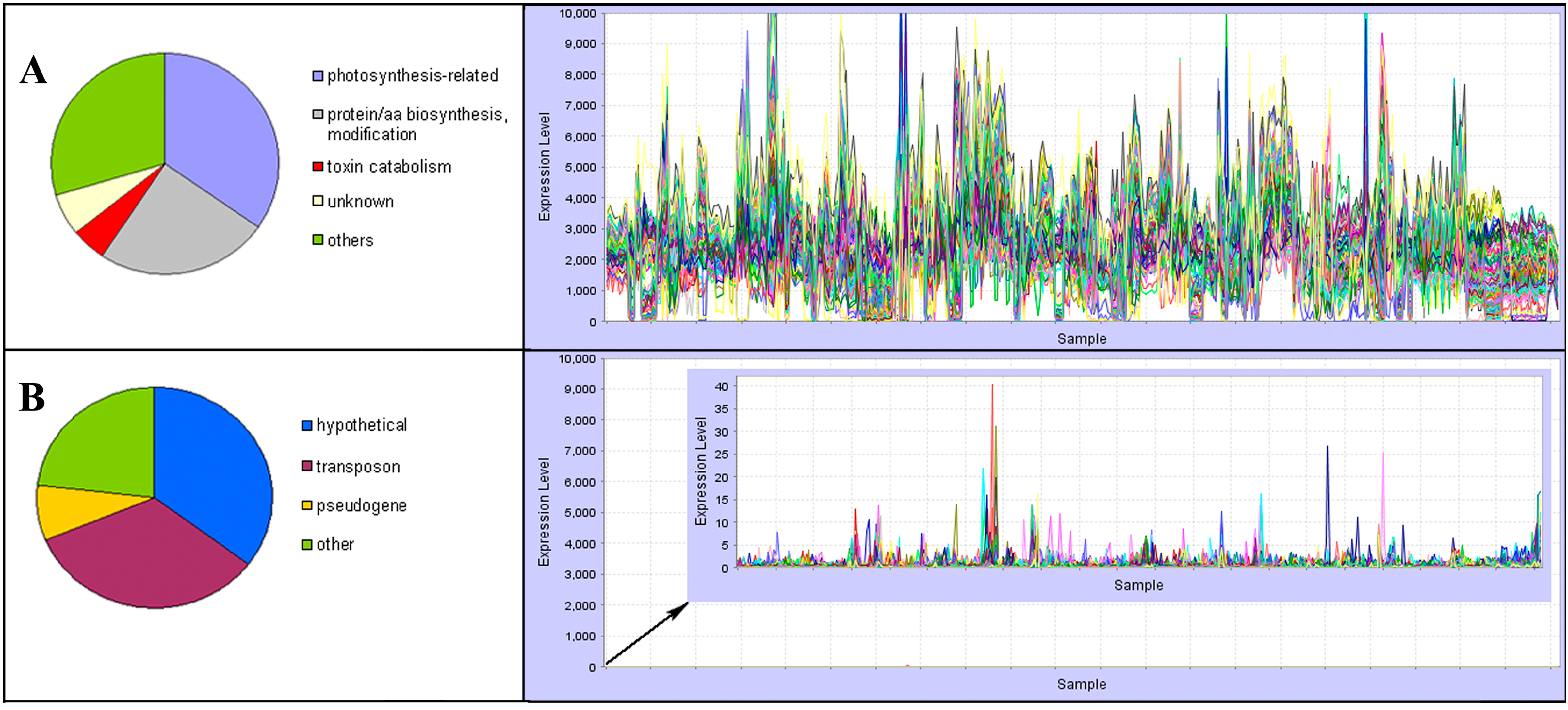

Supplement: Additional file 3 — Functional assignments and expression profiles of the 100 genes with (A) the highest expression (maximum mean), and (B) the lowest expression (minimum mean). [file 1471-2229-8-99-S3.tiff]
